# Targeting of Bacteria Using Amylase-Degradable, Copper-Loaded Starch Nanoparticles

**DOI:** 10.3390/antibiotics15010056

**Published:** 2026-01-04

**Authors:** Nathan A. Jones, Usha Kadiyala, Benjamin Serratos, J. Scott VanEpps, Joerg Lahann

**Affiliations:** 1 Program in Macromolecular Science & Engineering, University of Michigan, Ann Arbor, MI 48108, USA; 2 Department of Emergency Medicine, University of Michigan, Ann Arbor, MI 48108, USA; 3 Department of Materials Science & Engineering, University of Michigan, Ann Arbor, MI 48108, USA; 4 Department of Biomedical Engineering, University of Michigan, Ann Arbor, MI 48108, USA; 5 Weil Institute for Critical Care Research and Innovation, Ann Arbor, MI 48108, USA; 6 Biointerfaces Institute, University of Michigan, Ann Arbor, MI 48108, USA; 7 Department of Chemical Engineering, University of Michigan, Ann Arbor, MI 48108, USA

**Keywords:** starch nanoparticles, copper nanoparticles, *Staphylococcus aureus*, *Bacillus subtillis*, electrohydrodynamic jetting, metabolically responsive, amylase

## Abstract

**Background/Objectives:** The treatment of bacterial infections is complicated by emerging antibiotic resistance. This paper identifies a novel approach with a nanoparticle that targets bacterial surface charge and is responsive to the nutrient environment (i.e., glucose) and presence of metabolically active bystander species (i.e., amylase secretion) within microbial communities. **Methods:** Thus, metabolically responsive composite nanoparticles (440 ± 58 nm) were fabricated via electrohydrodynamic jetting of a cationic starch polymer incorporating 5–7 nm copper nanoparticles (0.3 wt%). Starch was selected as the base polymer, as it is a common carbon source for amylase-producing bacterial communities, in particular under glucose-limited growth conditions. **Results:** The resulting positively charged particles effectively associated with Gram-positive *Staphylococcus aureus*, forming co-aggregates with bacterial cells and exhibiting antibacterial activity tenfold greater than free copper nanoparticles. In co-cultures of *S. aureus* and the amylase-producing bystander species, *Bacillus subtilis*, enzymatic degradation of the copper–starch nanoparticles increased antibacterial activity against *S. aureus* by 44%. **Conclusions:** This work highlights the potential for metabolically regulated particles as a novel paradigm for selective, narrow-spectrum antibacterial therapies that exploit ecological interactions within microbial communities.

## 1. Introduction

Despite the widespread availability of small molecule antibiotics, bacterial infections remain a major cause of morbidity, mortality, and healthcare costs within hospital settings [1,2]. Approximately 3–13% of patients acquire an infection while being managed for a different condition, and incidence increases to nearly 50% for high-risk groups including newborns, immunocompromised patients, and those in intensive care units [3,4,5,6,7]. In the United States alone, antibiotic-resistant bacteria are involved in more than 2 million infections, with approximately 23,000 deaths annually [7].

Although the drivers for emerging antibiotic resistance are complex and multifactorial [8], they can be summarized as resulting from evolutionary pressure on bacteria outside the intended target or environment. Consequently, new options are needed to prevent and treat infections while limiting emerging resistance. To this end, recent efforts have explored antibacterial polymers [9] and metal nanoparticles, such as those made of silver [10,11,12], copper [12,13,14], or zinc oxide [15,16]. Metal nanoparticles exhibit broad-spectrum antibacterial activity with lower potential for resistance due to multiple simultaneous mechanisms of action, including oxidative stress, membrane disruption, metabolic disruption, and mismetallation [10,11,16].

However, the clinical translation of these antibacterial technologies is limited due to challenges such as poor aqueous solubility, scalability of production, and concerns over colloidal stability. Continued advances in medical nanotechnology are being developed to address these barriers, enabling improved targeted, responsive, and effective nanoparticle systems for bacterial infection control [17].

Targeted antibacterial approaches can be broadly categorized into passive and active schemes. Passive targeting methods rely on non-specific material properties to increase antibacterial dosage and efficacy. Bacterial infections, like cancerous tumors, are associated with increases in vascular permeability, allowing the Enhanced Permeation and Retention (EPR) effect for appropriately sized nanoparticles delivered intravenously [18]. Electrostatic interactions offer an effective method for targeting pathogens. Most bacteria exhibit a negative surface charge under physiological conditions, enabling cationic nanoparticles and polymers to bind through multivalent electrostatic interactions [9,13,19]. These passive targeting approaches provide broad utility, including the ability to target polymicrobial infections [19].

Active targeting methods, in contrast, employ specific ligands, such as vancomycin [20], lectins [21], antibodies [22], or aptamers [23], which are conjugated to antimicrobial agents or nanoparticle carriers to selectively recognize and bind to pathogenic bacteria [17,24]. A related approach, termed secondary targeting, involves directing nanoparticles to immune cells such as macrophages or other leukocytes, which are recruited to the infection site [25,26].

Beyond targeting specificity, nanoparticles have been engineered to respond to physicochemical cues within the infection microenvironment. Local acidity, for example, can trigger the degradation of acid-sensitive polymers, such as poly(lactic-co-glycolic acid) (PLGA) [19,27], poly(caprolactone) (PCL) [28], or chitosan [13]. Some polymers become cationic in acidic environments, enhancing bacterial binding [9,19]. More recently, enzyme-sensitive nanoparticles have been prepared that decompose in the presence of bacterial phosphatase or phospholipase [29].

Starch has attracted research interest as a biocompatible, renewable matrix in composite antibacterial materials, where it serves as both a stabilizer and a carrier for metal nanoparticles such as copper, enabling controlled release and enhanced contact with microbial cells [30,31,32,33]. In starch–copper nanocomposites, the polysaccharide framework helps disperse and protect small CuNPs, leading to strong bactericidal effects against both Gram-positive (e.g., *Staphylococcus aureus*, *Bacillus subtilis*) and Gram-negative (e.g., *Escherichia coli*) bacteria, often at low (microgram per milliliter) concentrations. These systems typically show concentration-dependent growth inhibition, with more potent activity against staphylococci and *B. subtilis*. Their efficacy is frequently attributed to copper ion release, ROS generation, and direct nanoparticle–cell membrane interactions, making starch–copper composites promising candidates for antimicrobial coatings, wound dressings, and biomedical devices. Villanueva et al. developed multi-use antibacterial composite gels with high loading (2.5–7.5% *w*/*w*) of silica-capped CuNPs (125–938 nm) embedded in a starch matrix [33]. Dinda et al. prepared starch–copper nanocomposites (~4 nm, unknown loading) that demonstrated antibacterial efficacy against *S. aureus* and *Pseudomonas aeruginosa* with minimum inhibitory concentrations (MICs) of 60 µg/mL and 75 µg/mL, respectively [30]. Hasanin et al. developed myco-synthesized starch–copper nanocomposites (200 nm) doped with 9 nm copper nanoparticles at 0.24% *w*/*w*, with highly negative surface charge (−150 mV), and more limited efficacy (125 µg/mL MIC) against Gram-negative bacteria like *E. coli*, with higher efficacy against Gram-positive bacteria including *B. subtilis* (7.81 µg/mL MIC) [31]. More recently, Hou et al. evaluated the impact of starch modifications, including acetylation, esterification, and amination on the morphology and qualitative antibacterial efficacy of prepared starch–copper composites against *S. aureus* and *E. coli*, finding that aminated starch improved antibacterial efficacy presumably due to incorporation of cationic charges [32]. Starch can be digested rapidly (20–30 min) by amylase enzymes, which can be present in biological environments, such as in the mouth and small intestines, or secreted by certain types of bacteria. Starch degradation by environmental amylase has been used for metabolically responsive payload release [33,34], and bacterial amylase secretion can be similarly leveraged [35,36].

In an attempt to develop a targeted nanomedicine, we decided to design, synthesize, and validate an amylase-responsive, cationic, starch-based nanoparticle platform (CuStNPs) that electrostatically associates with bacteria, and rapidly releases antibacterial copper nanoparticles (CuNPs) in the presence of amylase, an enzyme released by certain bacterial species (target or bystander) in ecological neighborhoods under specific nutrient conditions [37,38]. Potential species include *Bacillus* spp., *Lactobacillus* spp., *Pseudomonas* spp., *Vibrio* spp., and *E. coli* [39]. The composite CuStNPs allow for dispersion of hydrophobic CuNPs in aqueous media by providing colloidal stability within a cationic starch matrix, which may facilitate translation of CuNPs to clinical applications. This work introduces a distinct and novel paradigm of targeted antibacterial therapy that exploits vulnerabilities related to the metabolic ecology of microbial communities via secreted enzymes like amylase, which could be modified for alternative payloads or enzyme-substrate pairs.

## 2. Results & Discussion

### 2.1. Particle Preparation and Characterization

Electrohydrodynamic (EHD) jetting of particles and fibers has been extensively studied by our group [19,40,41,42,43,44], and others [45,46,47], employing various polymer compositions such as poly(lactic-co-glycolic) acid [42], acetal dextran [43,44], and stimuli-responsive polymers [40,41]. Starch is less commonly used in the fabrication of nanoparticles due to the inherent crystallinity of amylose, which promotes uncontrolled aggregation of prepared nanostructures [48]. However, successful approaches have been achieved through chemical modification and cross-linking of starch [49,50,51,52,53]. Starch is an interesting low-cost biocompatible material for metabolically responsive antibacterial applications because it is a common metabolite that can be degraded by amylase enzymes, which are produced by certain bacterial species [37,38,39].

**Figure 1:**
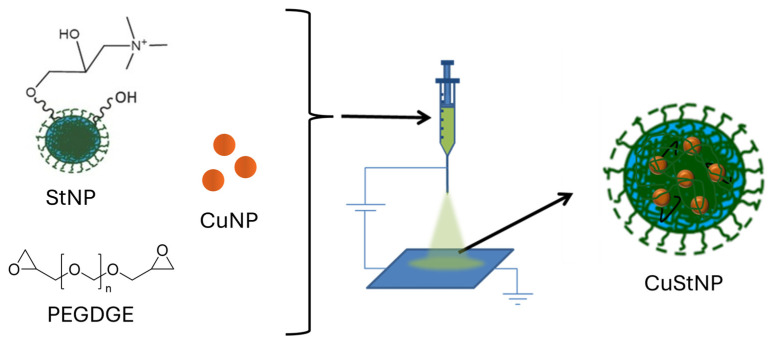
Schematic of CuStNP preparation via EHD jetting.

In this work, we use research-grade starch nanoparticles (StNPs), which were further modified to introduce cationic functionality [34]. These cationic StNPs were cross-linked following EHD jetting. Incorporation of other components into the jetting solution allows their encapsulation within the starch matrix. Specifically, 5–7 nm CuNPs were dispersed into the jetting solution, forming a semi-stable Pickering-type emulsion upon vortexing. This dispersion remained stable throughout jetting and deposition onto metal collection plates, prior to thermal curing and final CuStNP collection (Figure 1).

**Figure 2:**
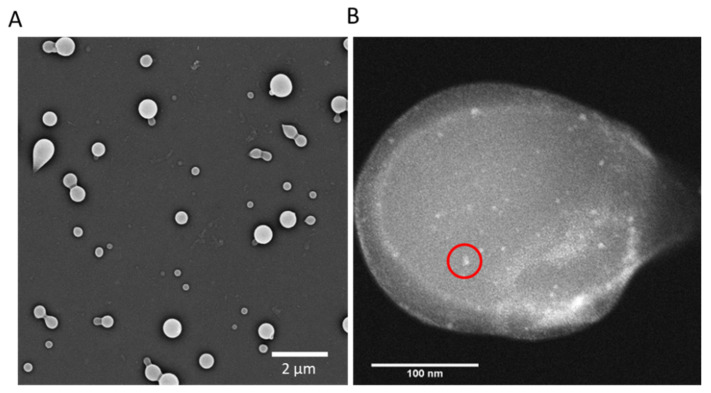
(**A**) SEM and (**B**) TEM images of CuStNP, showing high contrast 5–7 nm CuNPs (example circled) distributed throughout the larger StNP.

Representative SEM images (Figure 2A) show the spherical morphology and uniform size distribution of the resulting composite CuStNPs, while TEM (Figure 2B) demonstrates the homogeneous distribution of CuNPs within the larger starch matrix. Dynamic light scattering measurements indicated an average hydrodynamic diameter of 440 ± 58 nm. Comparison with size analysis by ImageJ v.2.0.0 from SEM [54], which showed a dry particle size of 193 ± 91 nm, suggests a swell ratio of approximately 2.3 ± 0.5. Particle surface charge was confirmed as cationic with a zeta potential of +22.7 ± 1.3 mV. Swelling of the particles is indicative of their “softness”, which allows for highly effective multivalent binding.

The copper content of CuStNP samples was quantified by inductively coupled plasma optical emission spectroscopy (ICP-OES) using a calibration curve made with copper ICP standards (Supplementary Figure S1; R^2^ > 0.99). Samples prepared in tryptic soy broth (TSB) at concentrations of 1, 2, and 4 mg/mL yielded an average CuNP loading of 0.34 ± 0.05 wt% with a loading efficiency of 89.8 ± 7.5% (mean ± standard error, *n* = 3). This loading is comparable to the loading reported in the recent literature for similar systems [31].

### 2.2. Antibacterial Efficacy

Antibacterial activity was assessed against three Gram-positive species (*S. aureus*, *Staphylococcus epidermidis*, and *B. subtilis*) and two Gram-negative species (*E. coli* and *Klebsiella pneumoniae*). *S. aureus* and *S. epidermidis* were included as common human skin and wound pathogens frequently used in antibacterial screening. *E. coli* and *K. pneumoniae* were selected as clinically important Gram-negative species associated with gastrointestinal and opportunistic infections. *B. subtilis*, although not typically a human pathogen, was included due to its well-characterized physiology and its endogenous amylase production, which allowed us to evaluate the stability and antibacterial performance of the starch-based nanoparticles in the presence of amylolytic activity. Bacteria were exposed to serial dilutions of CuStNP dispersions corresponding to 2.1–34 µg/mL CuNP (0.5–8.0 mg/mL CuStNP). Control experiments using unloaded StNPs were performed with *S. aureus* and *E. coli* and showed minimal effects on bacterial growth, even at the highest tested concentrations (Supplementary Figure S2).

**Figure 3:**
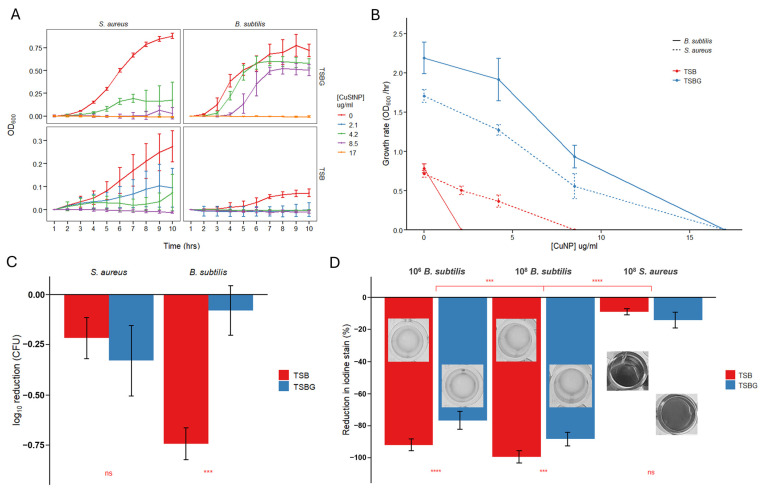
(**A**) Growth curves for *S. aureus* and *B. subtilis* upon exposure to escalating doses of CuStNPs in TSBG and TSB. (**B**) Comparison of the dose–response of the growth rate of *S. aureus* (dotted lines) or *B. subtilis* (solid lines) in TSB (red) or TSBG (blue). (**C**) Quantitative culture results after 7.5 h at 4.2 µg/mL CuNP dose (~1 mg/mL CuStNP), highlighting a significant increase in activity against starch-digesting *B. subtilis* in unsupplemented media, while activity against non-starch-digesting *S. aureus* is consistent independent of glucose supplementation. (**D**) Degradation of starch particles, as measured by reduction in iodine staining following an 8-h incubation with the noted cell types and initial counts. Inset images show representative black and white views of culture plate wells. Error bars represent standard deviation for three independent biological replicates. For statistical comparisons ns *p* > 0.05; *** *p* < 0.001; **** *p* < 0.0001.

Consistent with prior reports on copper nanoparticle activity [12,14,30,31,33], CuStNPs exhibited limited efficacy against Gram-negative bacteria (Supplementary Figure S3A,B), which has been hypothesized due to cell well composition resulting in stronger resilience to copper nanoparticles [30]. In contrast, strong antibacterial activity was observed against Gram-positive bacteria even at effective copper doses of 4.2 µg/mL for *S. aureus* and 8.5 µg/mL for *B. subtilis* and *S. epidermidis* (Figure 3A,C and Supplementary Figure S3C). This is an order of magnitude lower effective dose than previously reported for free CuNPs and other copper–starch composite systems [12,14,30,31,33]. Further mechanistic evaluation of potential reasons for this improved efficacy is explored below (Section 2.3). Dose–response curves were generated to compare the dose–response of each species’ growth for different media (Figure 3B). These results highlight a more significant reduction in growth rate constant for amylase-releasing *B. subtilis* absent supplemental glucose, with null growth at just 2.1 µg/mL CuNP vs. a dosage of 8.5 µg/mL CuNP for *S. aureus*, and further demonstrate complete eradication of bacterial growth with supplemental glucose at CuNP doses of 17 µg/mL.

We hypothesized that CuStNPs would exhibit greater antibacterial activity against *B. subtilis* than *S. aureus* due to the ability of *B. subtilis* to secrete amylase and enzymatically digest starch [39], thereby accelerating release of CuNPs. However, in TSB with supplemental glucose (TSBG), this trend was not observed in either growth curve analysis (Figure 3A) or quantitative culture (Figure 3C). There was a more potent response for *S. aureus* compared to *B. subtilis*, particularly at the 4.2 µg/mL CuStNP dose. However, when tested in unsupplemented TSB, the opposite trend emerged; *B. subtilis* growth was more strongly inhibited than *S. aureus*. This may partly reflect the reduced baseline growth of *B. subtilis* under glucose-limited conditions.

Nevertheless, the glucose-dependent differences suggest that *B. subtilis* metabolism and growth are sensitive to nutrient availability. Specifically, in carbohydrate-limited media, starch-degrading bacteria are expected to upregulate amylase expression to digest starch into monomer sugars for energy metabolism. This is analogous to the regulation of the lac operon in many bacterial species [38]. In this specific case, glucose limitation results in upregulation of amylase, which promotes enhanced nanoparticle degradation and CuNP release. Consistent with this hypothesis, CuStNPs exhibited increased antibacterial efficacy against *B. subtilis* in TSB (glucose limited) relative to TSBG (Figure 3B). Although a plausible explanation for the data, we cannot rule out the physical and chemical effects of glucose in the media, including osmolality and oxidative potential, as other contributors to this observation.

To complement the turbidometric growth curve analysis, quantitative culture studies were performed to directly measure bacterial killing (Figure 3C, Supplemental Figure S4). These results confirmed that *B. subtilis* killing is significantly greater (*p* = 0.00036) in unsupplemented TSB, whereas *S. aureus* showed no media-dependent difference (*p* = 0.213). At 4.2 µg/mL CuStNP, viable bacterial counts showed a maximum 1-log reduction with the following rank order of efficacy: *B. subtilis* in TSB > *S. aureus* in TSBG ≥ *S. aureus* in TSB > *B. subtilis* in TSBG (*p* < 0.001 by ANOVA with post hoc Tukey’s test, except between *S. aureus* media conditions).

Iodine staining confirmed starch degradation by *B. subtilis* but not *S. aureus* in both TSB and TSBG media following 8 h of incubation (Figure 3D). Starch degradation was significantly greater for *B. subtilis* in unsupplemented media (*p* = 0.000018 and *p* < 0.0001 for 10^8^ and 10^6^ cell counts, respectively), though more than 80% degradation occurred even in TSBG. At this late time point, apparent differences in degradation rate are not evident as increased cell density in TSBG cultures likely compensated for lower per-cell amylase expression.

### 2.3. Strong Interactions Between CuStNPs and S. aureus

**Figure 4:**
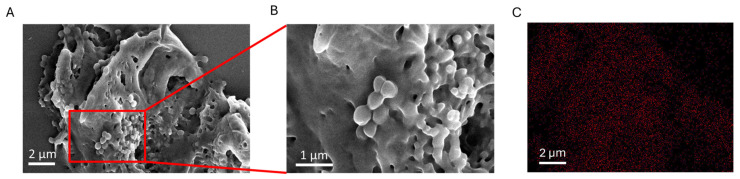
(**A**) Low and (**B**) high magnification SEM demonstrate “Clumping”, or heterogeneous aggregation of *S. aureus* exposed to CuStNPs. (**C**) Corresponding EDX mapping of copper (red) from SEM image in (**A**) showing high copper presence in vicinity of bacterial cells.

Given the unexpectedly high antibacterial efficacy observed with CuStNPs relative to their copper content, we hypothesized that the nanoparticles may interact directly with bacterial cells, facilitating localized copper delivery and increasing the effective local dose. Therefore, to test this, *S. aureus* cultures were incubated with 4.2 µg/mL CuStNP for 8 h, then prepared for imaging by SEM, confocal microscopy, and digital imaging (Figure 4).

Because these CuStNPs comprise hydrophilic polymers, they display nanogel-like properties and swell to more than twice their size after suspension in water. We propose that the cationic charge of the particles promotes strong multivalent interactions with the negatively charged bacteria, leading to the formation of agglomerates comprising bacteria and starch hydrogel nanoparticles. In SEM images, the dehydrated particles appear as a smooth coating enveloping the bacterial cells (Figure 4A,B), consistent with the adhesion hypothesis.

This association likely accounts for the enhanced antibacterial activity of the CuNPs. Although the overall copper dose (~4.2 µg/mL) is approximately an order of magnitude lower than in comparable studies [12,14], the CuNPs are effectively concentrated with the bacteria by the cationic hydrogel carrier. Elemental mapping by EDX confirmed the presence of copper localized to bacterial aggregates (Figure 4C). These findings support a mechanism where electrostatic association between CuStNPs and bacterial cells increases the effective local CuNP concentration, resulting in significantly enhanced antimicrobial potency relative to free CuNP dispersions.

### 2.4. Co-Culture of Amylase and Non-Amylase Producing Species

**Figure 5:**
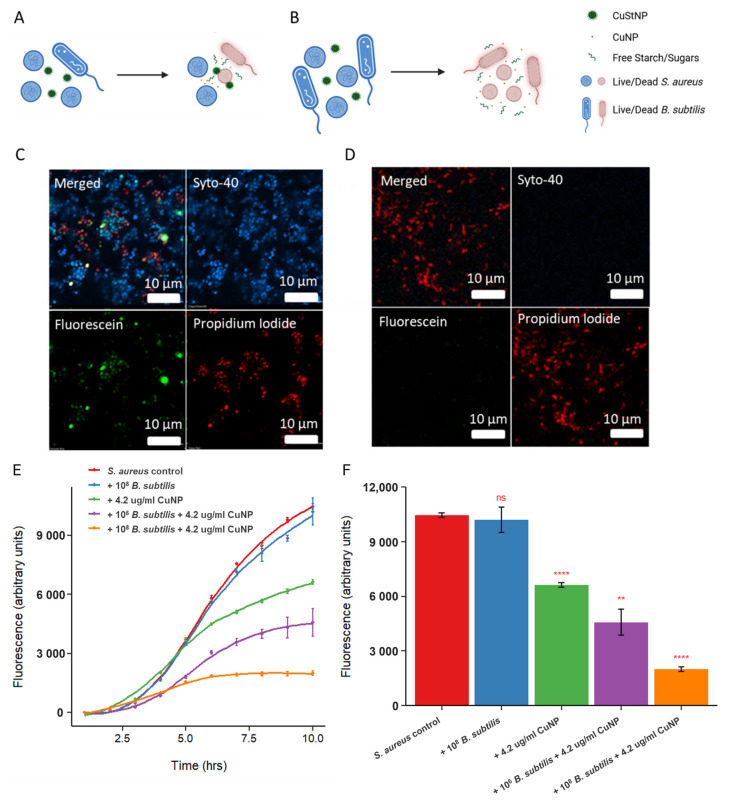
Bacterial co-culture viability and growth assays. (**A**) Schematic demonstrating the condition with low amount of rod-shaped *B. subtilis* relative to spherical *S. aureus*. Live bacteria are stained blue with Syto-40, while dead bacteria are stained red with propidium iodide, and the CuStNPs are stained green with fluorescein. (**B**) Schematic showing the condition with high amount of *B. subtilis* and resultant increased antimicrobial activity due to CuNP release. (**C**,**D**) Confocal micrographs showing the merged and individual channel images for conditions described in (**A**) and (**B**), respectively. (**E**) Bacterial growth curves upon exposure to CuStNP for GFP *S. aureus* in various co-culture conditions, showing synergistic dose–response based on the amount of *B. subtilis* in culture. (**F**) Bacterial fluorescence at 10 h from (**E**) with statistical comparisons to the *S. aureus* control. Error bars represent standard deviation for three independent biological replicates. For pairwise statistical comparisons to the *S. aureus* Control—ns *p* > 0.05; ** *p* < 0.01; **** *p* < 0.0001.

Bacteria-mediated starch digestion, via amylase production, provides a way to confirm our hypotheses of the amylase-responsive antibacterial effect (Figure 3) and starch mediated aggregation with *S. aureus* (Figure 4). Unlike *S. aureus*, *B. subtilis* enzymatically digests starch through the synthesis and excretion of amylase. Bacterial co-cultures containing 10^8^ *S. aureus* and either 10^6^ or 10^8^ *B. subtilis* were exposed to CuStNPs (4.2 µg/mL CuNPs). It was hypothesized that increasing numbers of *B. subtilis* would increase the availability of the CuNPs, resulting in higher overall antibacterial activity. Confocal microscopy with Live/Dead™ staining of bacterial cells was used to observe and determine antibacterial activity trends for various co-culture conditions. Expected results based on this hypothesis are illustrated in Figure 5A,B.

Experimentally, after 8 h of incubation, the co-culture seeded with low relative count (10^6^) *B. subtilis* showed a mixture of live and dead cells, with green fluorescently labeled CuStNPs interspersed (Figure 5C). In contrast, the co-culture seeded with 10^8^ *B. subtilis* was completely dead with green fluorescence dispersed, likely due to the degradation of the particles (Figure 5D). To support these observations, growth curves were charted for a GFP-producing *S. aureus* strain as a function of various co-culture conditions (Figure 5E), which indicate that the presence of *B. subtilis* had minimal effect on the growth of *S. aureus*, and increasing the amount of starch-digesting *B. subtilis* synergistically improved the antibacterial efficacy of the CuStNPs against *S. aureus*. Quantitatively, at 10 h, the number of *S. aureus* cells was reduced by 37% by treatment with 4.2 µg/mL CuNPs (*p* < 0.0001). The addition of an equivalent number (10^6^) of amylase-producing bystander *B. subtilis* lead to an additional 19% or total 56% reduction (*p* = 0.0037), while the addition of 10^8^ *B. subtilis* cells had an additional 44% or total 81% reduction (*p* < 0.0001).

**Figure 6:**
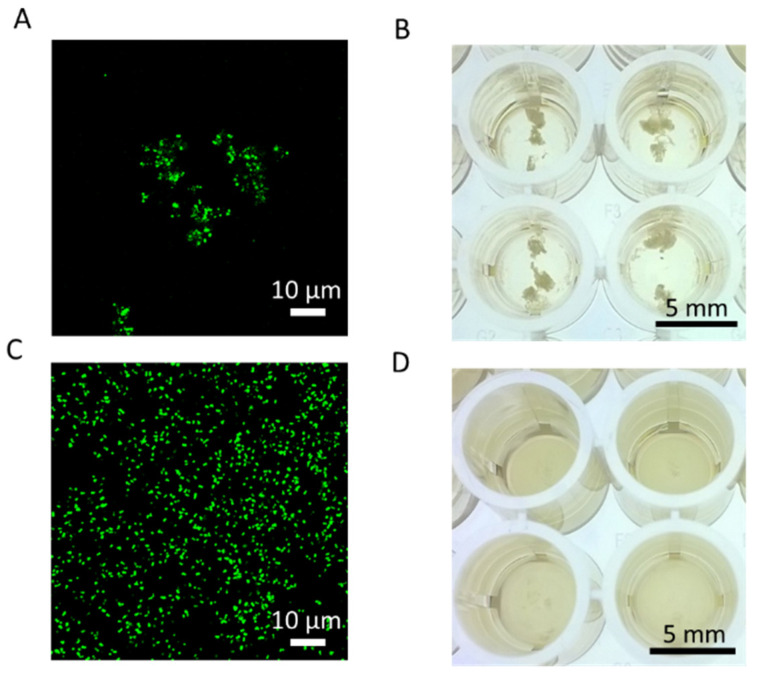
Aggregation in co-culture with fluorescently labeled CuStNPs shown by (**A**) confocal microscopy and (**B**) macroscopically in a 96-well plate for equal numbers of *B. subtilis* and *S. aureus* (106 initial inoculum each). (**C**) Confocal image and (**D**) photograph of 96-well plates showing minimal evidence of aggregation with 108 amylase-secreting *B. subtilis* and 106 *S. aureus*.

As noted, the cationic starch facilitated aggregation with negatively charged *S. aureus*. In co-culture samples with equivalent numbers of *S. aureus* and *B. subtilis*, this aggregation was observed microscopically using fluorescently labeled CuStNPs (Figure 6A), and macroscopically in the well plate (Figure 6B). In contrast, the co-culture sample with 100-fold excess of starch-digesting *B. subtilis* showed no evidence of aggregation (Figure 6C,D), presumably because the starch was digested by amylase.

### 2.5. Study Limitations and Future Directions

Our study demonstrates strong antibacterial activity of electrohydrodynamic jetted starch–copper nanocomposites against Gram-positive bacteria, but several limitations should be acknowledged. The current work focuses on planktonic growth inhibition in standard and glucose-free media, and future studies should extend to biofilm eradication, long-term stability of the composite under storage, and potential copper leaching under physiological conditions, as excessive copper ion release can raise biocompatibility concerns in biomedical applications [31]. Mechanical and barrier properties of the starch material (e.g., moisture sensitivity, mechanical strength) were not fully characterized here; evaluating these in future work would better inform the suitability of this material for wound dressings or food-contact surfaces. In addition, while efficacy against *S. aureus*, *S. epidermidis*, and *B. subtilis* is promising, broader testing against multidrug-resistant clinical isolates and in vivo models with amylase secretion (e.g., skin infection or wound healing models) will be essential to assess translational potential and safety profile.

## 3. Conclusions

In this study, we report the development of a novel composite starch nanoparticle platform prepared by reactive EHD jetting. The resulting particles, loaded with CuNPs, exhibited potent antibacterial activity against Gram-positive bacteria, including *S. aureus*, *S. epidermidis*, and *B. subtilis*. Antibacterial efficacy was improved under conditions favoring starch degradation, specifically in the presence of starch-digesting microbial communities (via amylase) and in media lacking glucose supplementation, demonstrating that nanoparticle composition can be leveraged to target therapeutic activity to specific metabolic environments.

The cationic charge of high-swelling CuStNPs facilitates strong electrostatic association with bacterial cells, localizing CuNPs at the bacterial surface and thereby increasing their effective potency. Co-culture experiments further revealed the potential for synergistic antibacterial effects in mixed bacterial populations containing starch-digesting species, as is relevant to natural microbiomes such as in the lower digestive tract.

Future work will investigate detailed mechanisms underlying antibacterial activity and evaluate the incorporation of other therapeutics into the StNP platform, potentially enabling targeted and metabolically regulated drug delivery within the gastrointestinal environment, wound dressings, or medical contact surfaces.

## 4. Materials and Methods

### 4.1. Materials

All chemicals were reagent-grade and purchased from Sigma Aldrich (St. Louis, MO, USA) except as noted. These include tryptic soy broth (TSB), glucose, glycidyltrimethylammonium chloride (GTAC), poly(ethylene glycol) diglycidyl ether (MW = 5k) (PEGDGE), sodium hydroxide, 2-propanol, and ethanol. Copper nanoparticles dispersed in organic phosphate ester, sized 5–7 nm (Product # 0851HN), were purchased from Sky Spring Nanomaterials (Houston, TX, USA). Research-grade starch nanoparticles (StNP) were provided by EcoSynthetix Inc. (Burlington, ON, Canada) [34].

### 4.2. Bacterial Strains, Media, & Growth Conditions

The bacterial strains used in this study were methicillin-resistant *S. aureus* COL [55], a green fluorescing protein (GFP) producing *S. aureus* strain (SH1000/pCM29) [56], *B. subtilis* PC194 [57], *S. epidermidis* 1457 [58], *E. coli* UTI89 [59], and *K. pneumoniae* LM21 [60]. Glycerol stocks of all strains maintained at −80 °C were plated on tryptic soy agar, cultured overnight at 37 °C and stored at 4 °C. Single colony inoculates were grown in tryptic soy broth with or without 1% glucose *w*/*v* (TSBG or TSB, respectively) under shaking conditions until optical density at 600 nm (OD_600_) reached 0.4–0.6 (mid-log), prior to dilution to OD_600_ of 0.01 for planktonic growth curves and quantitative culture studies.

### 4.3. Preparation & Characterization of Copper–Starch Nanoparticles

#### 4.3.1. Electrohydrodynamic Jetting of Copper Starch Nanoparticles

Electrohydrodynamic (EHD) jetting was performed using StNPs to control particle size, morphology, drug loading, and incorporate additional functionalities. Initial tests using StNP dispersions in water were unsuccessful as the solution would drip; substitution of up to 20% ethanol (*v*/*v*) lowered solution surface tension, improving jetting stability and yielding nanoparticles.

Copper–starch composite nanoparticles (CuStNPs) were prepared using EHD jetting as illustrated by the schematic in Figure 1. Cationic StNPs were prepared by chemically modifying research-grade StNPs, as reported previously [34]. The jetting formulation contained 10% (*m*/*v*) cationic StNP, 3% (*m*/*v*) PEGDGE cross-linker, and ~0.5% (*v*/*v*) of a 5–7 nm CuNP solution, dispersed in a solution of 80:20 deionized water/ethanol. Samples were vortexed to disperse hydrophobic CuNPs, creating a Pickering emulsion that remained visually stable over 24 h. EHD jetting was performed immediately after preparation of the jetting solution, using a voltage of ~12 kV, a pump speed of 0.05 mL/hr, and a jetting needle to collection plate distance of ~30 cm. Collected plates were subsequently cured in a 50 °C oven for 72 h and prepared CuStNPs were collected as a dry aggregate powder. Fluorescent CuStNPs were prepared by the same protocol, with substitution of 10% (*w*/*w*) of the cationic StNPs with particles additionally modified with covalently linked fluorescein (i.e., 1% *m*/*v* fluorescent cationic StNPs, 9% cationic StNPs *m*/*v* in the jetting formulation) [34]. To our knowledge, EHD jetting has found limited application in the preparation of starch particles with research focus on fibers and films [61], and just one recent example that evaluated methods for preparation of crystalline starch particles [48].

#### 4.3.2. Particle Size and Charge Characterization

Nanoparticle samples were dispersed in 0.01 M phosphate-buffered saline (PBS) solution at a concentration of 2.5 × 10^−4^ g/mL and characterized by dynamic light scattering (DLS) and electrophoretic light scattering (ELS) for zeta potential determination using a Malvern ZetaSizer (Malvern Panalytical, Malvern, UK). DLS measurements were performed at 25 °C with using automatic attenuation and measurement position selection. Zeta potential was determined by measuring electrophoretic mobility in folded capillary cells (Malvern Panalytical, Malvern, UK) via the Smoluchowski approximation. All samples were adjusted to pH 7.4 and measured in triplicate, allowing for calculation of average particle size and charge and estimates of error [62]. Particle size was also quantified from five representative scanning electron micrographs, each containing 50–100 particles per image, using the ImageJ v.2.0.0 particle analysis tool. To account for measurement variability, images were processed using three different sensitivity thresholds, and the resulting datasets were combined. All measurements were manually reviewed to correct for instances in which particle aggregates appeared, ensuring that only individual particles were included in the final size distribution.

#### 4.3.3. Electron Microscopy

Scanning electron microscopy (SEM) imaging was performed using a Quanta SEM (FEI, Hillsboro, OR, USA) with beam voltage of 3 kV and a magnification of 10,000× and a NOVA SEM (FEI, Hillsboro, OR, USA) with beam voltage of 5 kV, 0.4 nA, and at a magnification of 10,000×. Energy-Dispersive X-ray Spectroscopy (EDX) analysis was conducted to determine atomic mapping of copper to correspond with captured SEM images. Samples were mounted onto aluminum Scanning Electron Microscopy (SEM) stubs using carbon adhesive tabs and allowed to dry completely. The samples were then sputter-coated with a ~10–20 nm gold layer using an SPI-Module Carbon/Sputter Coater (West Chester, PA, USA) operated under argon at 18 mA for 120 s. Gold coating was used to improve sample conductivity and minimize charging during electron beam exposure. Transmission electron microscopy (TEM) imaging was performed using a JEOL 2100 (JEOL, Tokyo, Japan) probe-corrected analytical microscope with an accelerating voltage of 100 kV, a zirconated tungsten (100) thermal field emission tip filament, with gun vacuum at ~10^−9^ torr and column vacuum at ~10^−7^ torr (Michigan Center for Materials Characterization). For these images, nanoparticle powder was applied directly onto carbon-coated copper TEM grids by gently tapping a small amount of the dry powder over the grid surface. Excess material was removed by tapping the grid before loading into the microscope.

#### 4.3.4. Inductively Coupled Plasma—Orbital Emission Spectroscopy (ICP-OES) Evaluation of Copper Loading

ICP-OES measurements were performed using a Perkin-Elmer Optima 2000 DV (Waltham, MA, USA) with Winlab 6.4 software (MA, USA). Samples were doped with 1 ppm yttrium as a standard prior to degradation by addition of 5% (*w*/*w*) nitric acid. All CuStNP samples were prepared at mass concentration of 2 mg/mL in TSB and calibrated against a copper ICP standard (Sigma Aldrich, St. Louis, MO, USA). Three independent replicates were measured for each sample.

### 4.4. Bacterial Culture Studies

#### 4.4.1. Bacterial Growth Curves

CuStNP suspensions were prepared by vortexing 4.0 mg/mL CuStNPs into TSB or TSBG for 5 min. The final CuStNP concentrations were serial dilutions of the original stock solution. To compare to previously reported data on copper nanoparticles, concentrations are shown in terms of the CuNP concentration, as determined by the ICP loading study. The maximum concentration used in this study (25 µg/mL CuNPs, equivalent to ~4.0 mg/mL CuStNP, based on calculated loading of approximately 0.37% (*w*/*w*) CuNPs) was comparable to previously reported CuNP data [12,14]. Bacterial growth was assessed by measuring OD_600_ (Perkin Elmer Enspire multimode plate reader, Waltham, MA, USA) hourly over 10 h in the presence of CuStNPs. Dose–response curves were generated from the averages of three independent replicates. To compare individual growth curves we calculated the growth rate constant as the slope of the initial linear portion (i.e., exponential phase) of the log_2_(OD_600_) vs. time data determined by linear regression [15]. For co-culture experiments, *S. aureus* SH1000, transformed with a GFP-producing plasmid (pCM29), was used with *B. subtilis*. *S. aureus* growth was independently assessed by tracking green fluorescence (excitation 480 nm/emission 520 nm). Error estimates were calculated as the standard deviation from three independent replicates.

#### 4.4.2. Quantitative Culture

Bacterial cultures were exposed to 4.2 µg/mL CuNP dosage of CuStNPs (0.8 mg/mL) for 8 h, wherein cultures were serially diluted 10-fold in DI-H_2_O and plated on tryptic soy agar and allowed to grow for an additional 8–12 h for *B. subtilis* and 18–20 h for *S. aureus* prior to colony enumeration. The experiment was run in triplicate to allow for calculation of average logarithmic reductions and standard deviations. Statistical comparisons were evaluated using a two-tailed unpaired Student’s *t*-test.

#### 4.4.3. Iodine Test for Starch Degradation

Bacterial cultures were exposed to 4.2 µg/mL CuNP dosage of CuStNPs (0.8 mg/mL) for 8 h, mixed 50:50 with 1 M iodine solution to evaluate the starch concentration in solution. Sample absorbance readings were taken at 580 nm [63].

#### 4.4.4. Confocal Microscopy

Confocal microscopy was used to visualize bacteria-CuStNP interactions and evaluate bacterial viability in co-culture experiments. For bacteria-CuStNP interactions, fluorescein-labeled CuStNPs (dose 4.2 µg/mL CuNPs) were incubated with 10^8^ *S. aureus* or *B. subtilis* cells for 8 h. Samples were then transferred to confocal grade 8-well chambered coverglass wells (ThermoFisher 155411PK, Waltham, MA, USA) and viewed using a Nikon (Tokyo, Japan) A1RSi confocal laser scanning microscope, equipped with a CFI Plan Apo Lambda 100X oil objective with NA = 1.45 (Nikon, Tokyo, Japan). The excitation wavelength for fluorescein was 488 nm and imaging was captured with a fluorescein isothiocyanate (FITC) filter with emission 490–525 nm. For bacterial viability assessment of co-cultures, 10^8^ *S. aureus* cells were incubated with either 10^6^ or 10^8^ *B. subtilis* cells and fluorescein-labeled CuStNPs (dose 4.2 µg/mL CuNPs) for 8 h and stained using the LIVE/DEAD BacLight bacterial viability kit, (Molecular Probes Inc, Eugene, OR, USA). Samples (20 µL) were spotted onto confocal grade 8-well chambered coverglass wells (ThermoFisher 155411PK, Waltham, MA, USA) for imaging. The excitation wavelengths of the LIVE (SYTO 40) and DEAD (propidium iodide) dyes are 420 nm and 561 nm, respectively. Visualization was made by overlaid images with emission spectra for the live cells, dead cells, and CuStNPs of 435–485 nm, 570–620 nm, and 490–525 nm, respectively.

## Data Availability

The raw data supporting the conclusions of this article will be made available by the authors on request.

## Supplementary Materials

The following supporting information can be downloaded at https://www.mdpi.com/article/10.3390/antibiotics15010056/s1: Figure S1: Evaluation of copper loading by ICP-OES; Figure S2: Unloaded StNPs; Figure S3: Additional species; Figure S4: Dose comparison.

## Abbreviations

The following abbreviations are used in this manuscript:

EHD	Electrohydrodynamic jetting
StNPs	Starch Nanoparticles
CuNPs	Copper Nanoparticles
CuStNPs	Cupper Starch Nanoparticles
SEM	Scanning electron microscopy
TEM	Transmission electron microscopy
ICP-OES	Inductively Coupled Plasma—Orbital Emission Spectroscopy
TSB	Tryptic soy broth
TSBG	Tryptic soy broth with glucose
EDX	Energy-Dispersive X-ray Spectroscopy
