# Supplementary material for: Targeting of Bacteria Using Amylase-Degradable, Copper-Loaded Starch Nanoparticles
Source: Antibiotics (Basel). 2026 Jan 4;15(1):56. doi: 10.3390/antibiotics15010056 (PMC12838110; doi:10.3390/antibiotics15010056)
Supplement: Supplementary file 1 [file antibiotics-15-00056-s001.zip › antibiotics-4013212-supplementary.pdf]

Targeting of Bacteria Using Amylase-Degradable, Copper-Loaded Starch Nanoparticles

Nathan A. Jones[1], Usha Kadiyala[2], Benjamin Serratos[3], J. Scott VanEpps[1,2,4,5,6,*], and Joerg Lahann[1,3,4,6,7,*]

1 Program in Macromolecular Science & Engineering, University of Michigan, Ann Arbor, MI 48108, USA
2 Department of Emergency Medicine, University of Michigan, Ann Arbor, MI 48108, USA
3 Department of Materials Science & Engineering, University of Michigan, Ann Arbor, MI 48108, USA;
4 Department of Biomedical Engineering, University of Michigan, Ann Arbor, MI 48108, USA
5 Weil Institute for Critical Care Research and Innovation, Ann Arbor, MI 48108, USA
6 Biointerfaces Institute, University of Michigan, Ann Arbor, MI 48108, USA
7 Department of Chemical Engineering, University of Michigan, Ann Arbor, MI 48108, USA
** Correspondence: jvane@med.umich.edu (J.S.V.); lahann@umich.edu (J.L.)*

Supplemental:

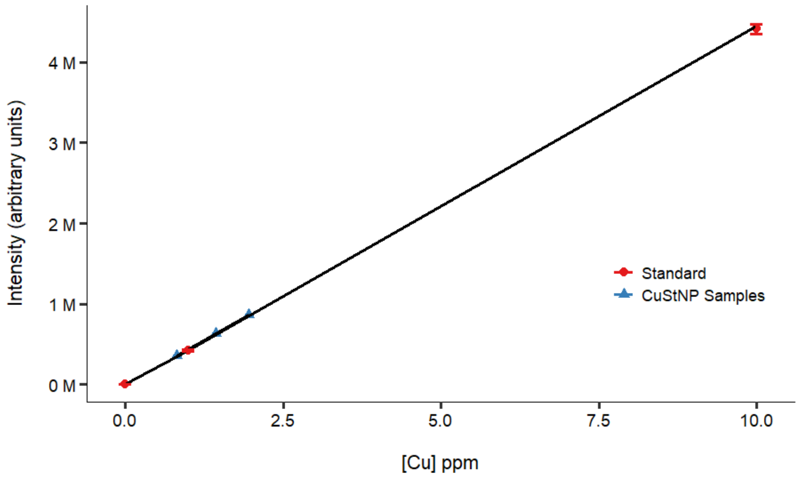

Figure S1: Evaluation of copper loading by ICP-OES. Samples prepared at 1, 2, and 4 mg/ml were compared to ICP standards to determine a loading of approximately 0.34%.

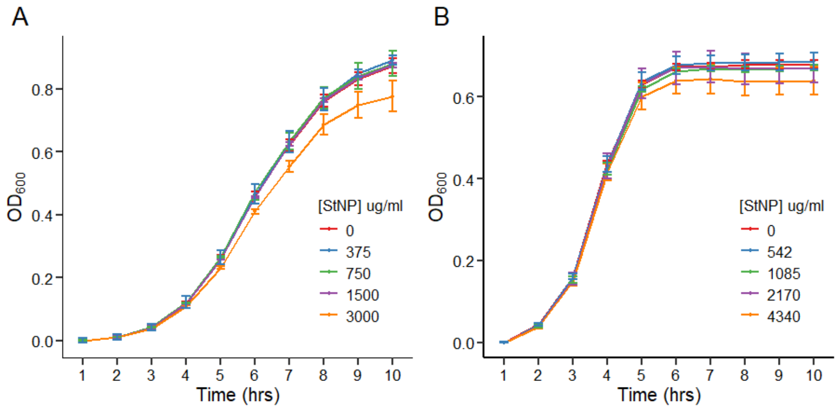

Figure S2: Unloaded StNPs. Growth curves of *S. aureus* (A) and *E. coli* (B) for escalating doses of unloaded cationic StNPs. Marginal toxic effect was only seen at the highest measured doses (~3000 µg/ml and ~4340 µg/ml for *S. aureus* and *E. coli*, respectively).

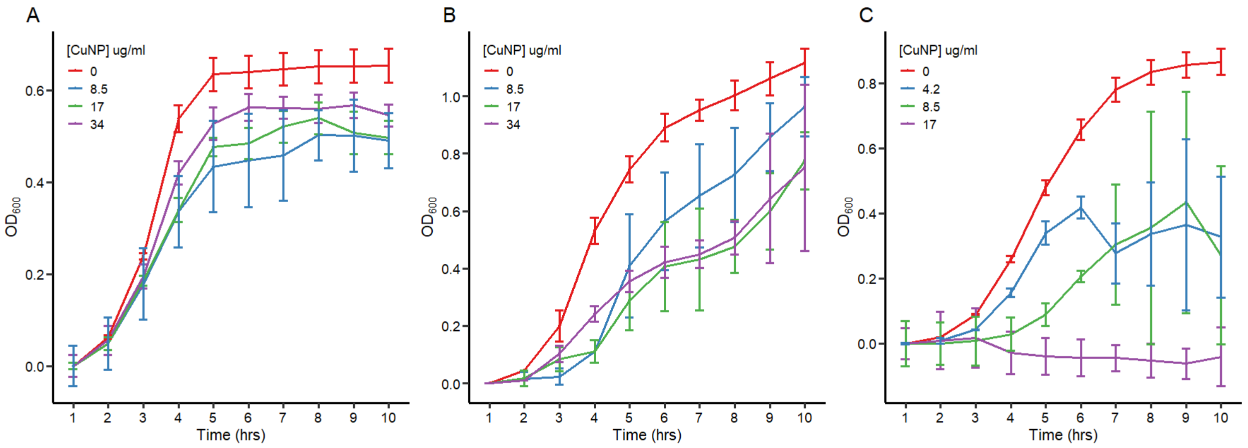

Figure S3: Additional species. Growth curves for escalating doses of CuStNP in TSBG with Gram-negative species (A) *E. coli* and (B) *K. pneumoniae* and Gram-positive species (C) *S. epidermidis.*

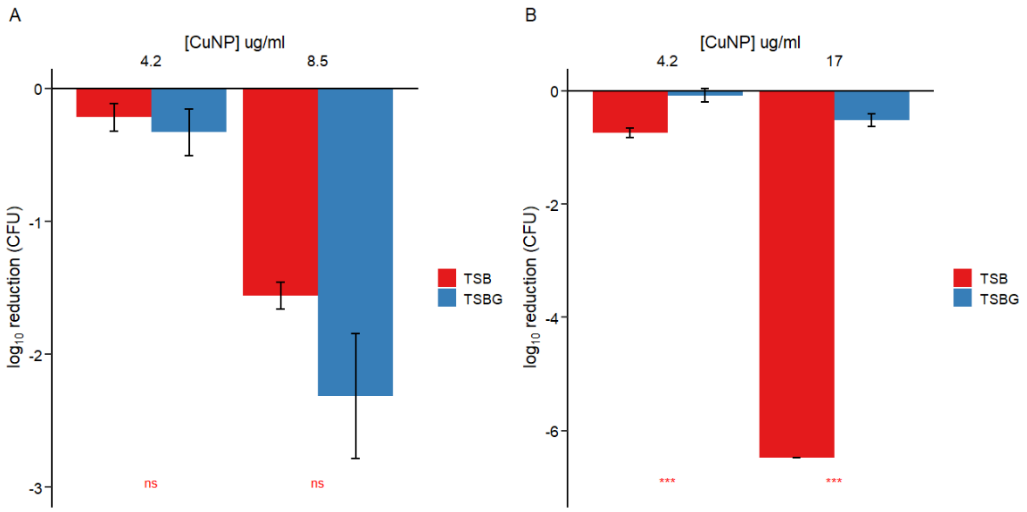

Figure S4: Dose comparison. Comparison of quantitative culture results at 4.2 g/ml doses and at higher doses, emphasizing the greatly enhanced efficacy of the CuStNPs against (A) *S. aureus* and (B) *B. Subtilis* in media without additional glucose. Error bars represent standard deviation for three independent biological replicates. For statistical comparisons ns $p>0.05$; * $p<0.05$; ** $p<0.01$; *** $p<0.001$; **** $p<0.0001$.
